# Focal Experimental Injury Leads to Widespread Gene Expression and Histologic Changes in Equine Flexor Tendons

**DOI:** 10.1371/journal.pone.0122220

**Published:** 2015-04-02

**Authors:** Else Jacobsen, Andrew J. Dart, Takamitsu Mondori, Neil Horadogoda, Leo B. Jeffcott, Christopher B. Little, Margaret M. Smith

**Affiliations:** 1 Research and Clinical Training Unit, University Veterinary Teaching Hospital, University of Sydney, Camden, New South Wales, Australia; 2 Raymond Purves Bone and Joint Research Laboratories, Institute of Bone and Joint Research, Kolling Institute of Medical Research (University of Sydney) at Royal North Shore Hospital, St. Leonards, New South Wales, Australia; University Hospital of Modena and Reggio Emilia, ITALY

## Abstract

It is not known how extensively a localised flexor tendon injury affects the entire tendon. This study examined the extent of and relationship between histopathologic and gene expression changes in equine superficial digital flexor tendon after a surgical injury. One forelimb tendon was hemi-transected in six horses, and in three other horses, one tendon underwent a sham operation. After euthanasia at six weeks, transected and control (sham and non-operated contralateral) tendons were regionally sampled (medial and lateral halves each divided into six 3cm regions) for histologic (scoring and immunohistochemistry) and gene expression (real time PCR) analysis of extracellular matrix changes. The histopathology score was significantly higher in transected tendons compared to control tendons in all regions except for the most distal (*P* ≤ 0.03) with no differences between overstressed (medial) and stress-deprived (lateral) tendon halves. Proteoglycan scores were increased by transection in all but the most proximal region (*P* < 0.02), with increased immunostaining for aggrecan, biglycan and versican. After correcting for location within the tendon, gene expression for aggrecan, versican, biglycan, lumican, collagen types I, II and III, *MMP14* and *TIMP1* was increased in transected tendons compared with control tendons (*P* < 0.02) and decreased for *ADAMTS4*, *MMP3* and *TIMP3* (*P* < 0.001). Aggrecan, biglycan, fibromodulin, and collagen types I and III expression positively correlated with all histopathology scores (*P* < 0.001), whereas lumican, *ADAMTS4* and *MMP14* expression positively correlated only with collagen fiber malalignment (*P* < 0.001). In summary, histologic and associated gene expression changes were significant and widespread six weeks after injury to the equine SDFT, suggesting rapid and active development of tendinopathy throughout the entire length of the tendon. These extensive changes distant to the focal injury may contribute to poor functional outcomes and re-injury in clinical cases. Our data suggest that successful treatments of focal injuries will need to address pathology in the entire tendon, and that better methods to monitor the development and resolution of tendinopathy are required.

## Introduction

Tendon injuries represent a major problem for the both the professional and leisure sporting communities[1,2]. In particular, Achilles tendon injuries occur frequently in activities involving running and jumping[3,4]; shoulder tendon injuries are frequent in baseball[5] and racket sports and flexor tendon injuries commonly occur in young workers[6]. Current clinical treatments are limited, lengthy and often result in suboptimal outcomes, and repair of damaged tendons may or may not reduce the risk of re-injury[7,8]. Recurrent injury, despite apparently successful healing/repair, is a common complication following tendon tears, with reported re-rupture rates ranging from 3–9% in Achilles[9], 25% in hamstring[10,11], 6% in flexors[6] and up to 95% in the rotator cuff[12]. In addition to re-failure, chronic pain and dysfunction are reported by up to 40% of patients after rotator cuff repair[12].

The reasons for the remarkably poor outcomes and high recurrence rates after tendon injury remain unclear but have largely focussed on changes at the injury site (reviewed in [13]). Tendon healing, either within the tendon itself or at the bone-tendon junction, is slow and the resulting tissue is often fibrotic without recapitulating normal structure, even after a prolonged rehabilitation. This “tendon scar” does not have the same material properties and thus may be compromised under subsequent loading. In addition, adhesions between the healed tendon and surrounding tissue may limit normal excursion, leading to stiffness and dysfunction. Our previous findings in the ovine infraspinatus tendon[14] support an alternative hypothesis, that following focal injury, a widespread tendinopathy develops and it is this that leads to re-injury. In humans, pre-existing histopathologic tissue is highly prevalent in tendons of patients sustaining a spontaneous rupture[15] and ruptured Achilles tendons have a more degenerate histopathology than chronic pathological tendons[16]. This suggests not only that pre-existing degeneration is a predisposing factor to injury, but also that following a tear further degenerative change occurs. How far from the injury such pathology extends is unclear.

Recurrent tendon injury is also a significant clinical problem in athletic horses; incidence ranges from 10–40%[17], with approximately half of the horseracing injuries in Britain involving the superficial digital flexor tendon (SDFT). While 46% of tendon injuries in racehorses have been attributed to overstrain, the role of pre-existing tendinopathy from a previous undetected injury is unclear[18]. A previous diagnosis of tendinopathy is the highest risk factor for developing future tendinopathy (odds ratio 8.5, 95%CI 6.1–12)[19]. As in humans, tendon re-injury rates have been estimated as 53% by three years in a Scottish case-control study[20], and 70% of Japanese racehorses with tendon problems fail to reach their pre-injury performance level[21]. Dyson[22] suggested that the very high rate of SDFT re-injury in racehorses may, as in humans, be attributed to the poor quality of the repair tissue. However, biomechanical studies[23] have suggested that SDFT re-injury is most likely to occur adjacent to the initial injury, in the bordering “unaffected” tendon. This suggests that increased risk of re-injury may be attributed to the development of tendinopathy distant to the injury site. There is a paucity of data describing how far away from the initial lesion tendon is altered and how this may contribute to re-injury.

The pathogenesis of tendon degeneration is not well understood, although it is known to involve transient inflammation and synthesis of an abnormal tendon matrix with altered biomechanical properties[1,3,24]. Both the process of tendon degeneration and that of remodelling involve increased synthesis of extracellular matrix molecules and increased activity of the enzymes responsible for their turnover[25]. There are few studies that discriminate between tendon lesion repair, often involving early inflammation and tissue granulation[3], and the development of tendinopathy at sites distant from an acute injury[26]. The length of the equine SDFT provided an ideal model to determine the extent to which tendinopathy develops in the tissue around a local injury. A better understanding of the molecular mechanisms of post-injury tendinopathy and how this may differ with distance form the lesion may provide novel targets to improve the long-term outcome of tendon injury. The objective of this present study was hence to quantify the extent of the histologic and gene expression changes that occurred in adjacent tendon regions six weeks after experimental injury (partial transection) in the equine SDFT. We hypothesised that there would be widespread, degenerative histopathology throughout the tendon, not merely near the lesion site, and that there would be associated changes in gene expression of ECM components and the enzymes and inhibitors responsible for their turnover.

## Materials and Methods

### Ethics Statement

All animal procedures were approved by the University of Sydney Animal Care and Ethics Committee (protocol N00/11-2007/1/4739) and complied with their guidelines.

### Animal procedures including ultrasonography

The study used nine healthy Standardbred geldings aged 3–19 years purchased from local saleyards with normal forelimb SDFTs on physical and ultrasonographic examination (Phillips HD 11 Ultrasound; Phillips Electronics, North Ryde, NSW, Australia), and no signs of lameness at walk or trot. All horses had three months paddock rest prior to the start of the study; exercise history before this time was unknown.

**Fig 1:**
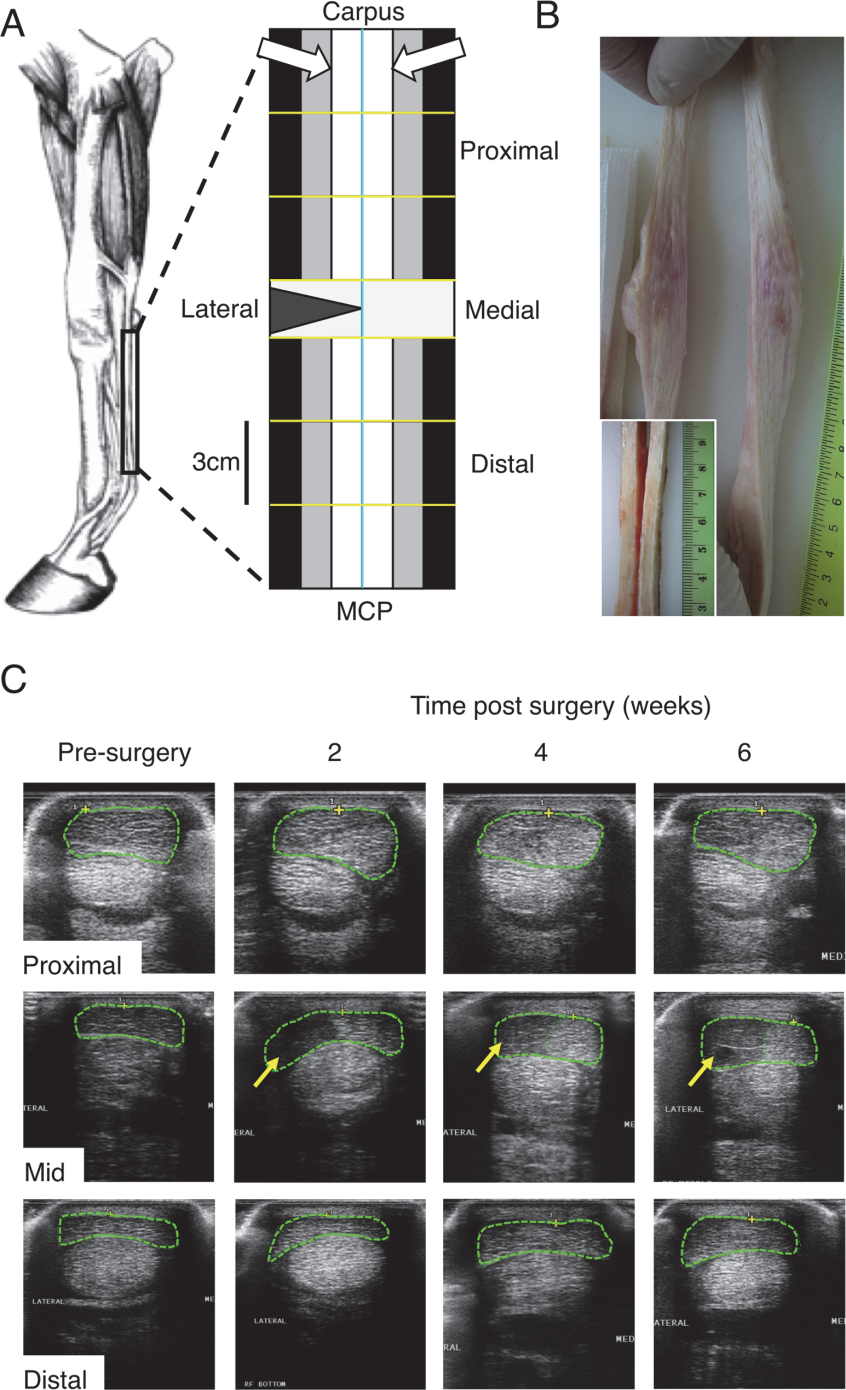
Transection details and ultrasound. A) Schematic diagram of the equine forelimb showing the metacarpal region of the superficial digital flexor tendon (SDFT; width not to scale) used in this study. The tendon was divided into 12 regions; longitudinally lateral and medial (cyan line) and the 1–4cm, 4–7cm and 7–10cm proximal and distal to the midmetacarpal (yellow lines; transection/lesion site indicated by the triangle). Each region was then further longitudinally divided into three portions for different analyses; gene expression (black), biomechanics (grey; not presented here) and histology (white). The histology sections are viewed from the face of the tendon indicated by the white arrows. B) Gross morphology of a representative transected tendon at harvest, six weeks after surgery presented at the same vertical scale (ruler shows cm) as in A) Inset is a control tendon at the lesion area for comparison. C) Ultrasonographs of the operated SDFT of one horse pre-surgery and two, four and six weeks post-transection at three indicated locations along the tendon. The SDFT is outlined with green dashes with areas of hypoechogenicity indicated with arrows.

Horses were randomly allocated (numbers drawn from a hat) to either the treatment or the control group. Six horses had the lateral 50% of one randomly selected forelimb SDFT transected in the mid-metacarpus (Fig 1A) under general anesthesia (treatment group). As there was concern these horses would offload the operated limb after surgery and because loading has been shown to induce changes in tendons[27], the contralateral SDFTs from these horses were not used as controls. The three remaining horses had sham surgery on one forelimb with the contralateral SDFT used as a non-operated control.

### Tendon surgery

Peri-operative antibiotic prophylaxis was provided with procaine penicillin (22mg/kg IM) (Propercillin, Troy Laboratories, North Ryde, NSW, Australia) and gentamicin sulfate (6.6mg/kg IV) (Gentam 100, Troy Laboratories). Horses were pre-medicated with xylazine hydrochloride (1.1mg/kg IV) (Xylazine, Troy Laboratories) and anesthesia was induced with ketamine hydrochloride (2.2mg/kg IV) (Ketamine, Troy Laboratories) and diazepam (0.1mg/kg IV) (Pamlin, Parnell Laboratories, North Ryde, NSW, Australia). Anesthesia was maintained with additional ketamine hydrochloride and xylazine hydrochloride as required. Analgesia was provided with butorphanol (8mg IV) (Torbugesic, Troy Laboratories) and phenylbutazone (4.4mg/kg IV) (Butasyl, Fort Dodge, North Ryde, NSW, Australia).

A 5cm skin incision was made on the palmar aspect of the mid-metacarpus and the SDFT isolated using hemostats. In six horses the lateral 50% by width of the isolated SDFT at the mid-metacarpal level was transected using a scalpel blade. Sham-operated horses had the SDFT isolated but not transected. The skin incision was closed with simple interrupted sutures and a bandage was applied. Phenylbutazone (2.2mg/kg orally BID) was continued for three days after surgery. Horses were kept in compatible groups of three in small yards (12 m x 12 m) with shelter available for six weeks. They were fed 2.5% (w/w) lucerne hay in divided feeds daily and had unlimited access to fresh water. Bandages and sutures were removed two weeks after surgery.

Ultrasonographic examination of the tendons (at the lesion site and up to 8cm proximal and distal to the lesion) was performed prior to surgery and at two, four and six weeks post-surgery. Horses were euthanased six weeks after surgery using an intravenous (jugular) overdose of sodium pentobarbitone (Lethabarb, Virbac, Milperra, Australia).

### Sample collection

Immediately following euthanasia the legs were shaved and aseptically prepared by using alternate scrubs of isopropyl alcohol and iodine to avoid contamination of the harvested tendons. A longitudinal skin incision extending from the carpal to metacarpophalangeal joint (Fig 1A) was made on the palmar aspect of the metacarpus and the skin reflected to either side. The SDFT was sharply transected proximally at the level of the accessory carpal bone and distally at the level of the metacarpophalangeal joint then removed and stored on ice in sterile saline soaked gauze in a plastic bag. Tendons were kept moist with sterile saline throughout the subsequent dissections (see below); these were completed within three hours of death. The partially transected SDFTs (n = 6), the sham-operated SDFTs (n = 3) and non-operated control SDFTs (n = 3) were all processed in an identical manner.

The paratenon was removed and each tendon was divided longitudinally into medial and lateral halves. Each half tendon was further divided into 6 x 3cm long samples (1–4cm, 4–7cm and 7–10cm proximal and distal to the transection site) making a total of 12 samples (not including the transection site). Each of these 12 samples were further longitudinally divided into three sections for (1) gene-expression, (2) histology/immunohistology and (3) biomechanics (Fig 1A). Tissue for RNA extraction was trimmed of all easily removable epitenon, then snap frozen in liquid nitrogen. Histology samples were placed in 10% (v/v) neutral buffered formalin. Tissue for biomechanical testing was wrapped in gauze soaked in isotonic saline, sealed in 2mL microtubes and stored at -20°C. Biomechanical testing was not performed as part of the present study.

### RNA isolation, reverse transcription and real time RT-PCR

Tendon samples were processed as previously reported for sheep infraspinatus tendon[14]. Briefly, weighed portions of tendon frozen in liquid nitrogen were pulverised to powder in a Dismembrator (Braun, Melsungen, Germany). Total RNA extraction was performed using TRIzol Reagent (Invitrogen, Melbourne, VIC, Australia), chloroform and RNeasy Mini Kits (Qiagen, Doncaster, VIC, Australia) with an on-column DNase step (RNase-Free DNase Set; Qiagen) according to the manufacturer’s instructions. RNA quantification was performed using SYBRGreen II (Cambrex) and 28S RNA standards (Sigma-Aldrich) in a microplate assay; the resultant fluorescent was determined using Synergy 2 microplate reader, (Biotek, Vermont, USA). RNA (1μg) was reverse transcribed into complementary DNA (cDNA) using an Omniscript RT Kit (Qiagen), random hexamers (Amersham, Buckinghamshire, UK) and RiboSafe RNase Inhibitor (Bioline, Alexandria, NSW, Australia). Real-time polymerase chain reaction (PCR) was performed in a Rotor-Gene 6000 (Corbett Life Science, Mortlake, NSW, Australia) using Immomix (Bioline), SYBRGreen I (Cambrex) and primers designed using MacVector software, version 7.2.2 (Accelrys, San Diego, CA, USA) (Table 1). All primers were purchased from Sigma Genosys (North Ryde, NSW, Australia). Standard curves (4-fold dilutions of stock equine tendon cDNA) were included with each run and expression (relative fluorescent units) determined for each gene using the Rotorgene software. Melt curves were obtained to verify a single amplification product.

**Table 1:** Primer sequences for real-time PCR of equine tendon cDNA.

Gene	Direction	Primer sequence (5’–3’)	Anneal (°C)	Product (bp)
*ACAN*	Forward	TCA GGT ATC CCA TCC ACT TGC C	57	105
	Reverse	CGT CGT AGG TCT CAT TGG TGT CAC	57	105
*ADAMTS4*	Forward	ATG TGG TCA CTA TTC CTG CGG G	58	137
	Reverse	AGG GCA TCA GCG TGT ATT CAC C	58	137
*ADAMTS5*	Forward	TTA CGA GAG AGG ATT TAT GTG GGC	56	217
	Reverse	CGC TTA TCT TCT GTG GAA CCA AAG	56	217
*BGN*	Forward	TGA TTG AGA ACG GGA GCC TGA G	56	143
	Reverse	TTT GGT GAT GTT GTT GGT GTG C	56	143
*COL1A1*	Forward	CCT GGC AAG AAC GGA GAT GAT G	59	140
	Reverse	CCA CTG AAA CCT CTG TGT CCC TTC	59	140
*COL2A1*	Forward	ACT TTC CAA TCC CAG TCA CGC	56	129
	Reverse	GAT TCT CTG GAC TTG ACC CGT CTG	56	129
*COL3A1*	Forward	GGA AGT TGC TGA AGG AGG ATG C	55	166
	Reverse	TGG AAT CTC TGG GTT GGG ACA G	55	166
*COMP*	Forward	GCA AAC AAT GAA CAG CGA CCC	56	129
	Reverse	TGG TAG CCA AAG ATG AAG CCC	56	129
*DCN*	Forward	CCA AAG TGC GAA AGT CTG TGT TC	54	138
	Reverse	CAG CAA TGC GGA TGT AGG AGA G	54	138
*FMOD*	Forward	GCT CCA TCT TGA CCA CAA CCA G	55	123
	Reverse	CCT TTC ATA GAA CTG CCC ACT TCC	55	123
*LUM*	Forward	AGG TGC GTT TAC TTT CCT CTT GG	55	239
	Reverse	GGT CAA TCT GGT TAT TCC GAA GG	55	239
*MMP3*	Forward	ATT CTG CTG TTA CTA TGC GTG GC	55	218
	Reverse	TTT CCT GTC ACC TTC AAC CCC	55	218
*MMP14*	Forward	ACC AGG TGA TGG ATG GAT ACC C	56	126
	Reverse	CCC AGT GCT TGT CTC CTT TGA AG	56	126
*TIMP1*	Forward	GGA TAC TTC CAC AGG TCG GAG AAC	59	234
	Reverse	CGT CCA CAA GCA ATC AGT GTC AC	59	234
*TIMP2*	Forward	ACT CTG GCA ACG ACA TCT ACG G	57	261
	Reverse	TCT TCT TCT GGG TGG CAC TCA G	57	261
*TIMP3*	Forward	AGG GGC TGA ACT ATC GGT ATC ACC	59	242
	Reverse	TGC TCA AGG GTC TGT GGC ATT G	59	242
*VCAN*	Forward	CCC ATC TCA CAA GCA TCC TGT C	55	123
	Reverse	TGC CAT CAG TCC AAC GGA AG	55	123

Primers were designed by MacVector 11.1.

### Histology

Histology procedures were performed at room temperature unless otherwise noted. Tendon tissue was fixed for 48 hours in 10% (v/v) neutral buffered formalin, softened for 48 hours in 10% (v/v) formic acid and 2.5% (v/v) formalin then transferred to 70% (v/v) ethanol for storage. Tendon samples were dehydrated starting with 70% (v/v) ethanol for at least 24 hours, then 80% (v/v) ethanol for five hours, 95% (v/v) ethanol for five hours and finishing with four changes of 100% (v/v) ethanol once every three hours. Tendon samples were cleared in methyl benzoate for three days then infiltrated with 1% (w/v) celloidin and 1.5% (w/v) trycresyl phosphate in methyl benzoate for three days then 5% (v/v) celloidin and 1.5% (v/v) trycresyl phosphate in methyl benzoate for three weeks. Specimens were rinsed with chloroform (three changes), infiltrated with paraffin wax (eight changes over four days) then embedded in paraffin blocks. Paraffin blocks were softened for six hours in 5% (v/v) formic acid, 45% (v/v) ethanol and 50% (v/v) glycerol on a cold plate then rinsed in cold water. Sections were cut at five microns using a Leica RM2255 rotary microtome (Leica Microsystems, Wetzlar, Germany) with Feather blades N35 (Arthur Bailey Surgico, Roselle, NSW, Australia). Sections were attached to Menzel-Glaser SuperFrost Ultra Plus slides (HD Scientific, Wetherill Park, NSW, Australia) and placed in an incubator at 85°C for at least 30 minutes, then kept at 55°C overnight. Serial sections from each of the tendon regions were stained with hematoxylin and eosin (H&E), picrosirius red (PSR) or toluidine blue using standard methods[14].

Sections were scored for histopathology as previously described for ovine infraspinatus tendon[14]. Coded sections were independently scored by three observers (EJ, TM and MMS), blinded to surgical group or tendon region, for intrafasicular cellularity (0–3), intrafasicular cell morphology (0–3), vascularity (0–3), interfascicular cell infiltration (0–3) and collagen fiber alignment (PSR under optimized polarized light; 0–3). The sum of these ordinal scores gave “histopathology score” (0–15), where lower scores were more normal. Toluidine blue sections were scored (0–3) for “proteoglycan score”, where zero is no proteoglycan visible and 3 is widespread staining for proteoglycan. Images of representative sections were captured using a light microscope (polarized for PSR sections), digital camera and Image Manager software (all from Leica Microsystems).

### Immunohistochemistry

Sections from lateral tendon proximal to the lesion were prepared from 2 randomly selected control and transected tendons (two regions per tendon: one adjacent and one most distant to the lesion), and pretreated with either hyaluronidase (Sigma #H3506, 1000U/mL for one hour at 37°C; slides for aggrecan) or chondroitinase ABC (Sigma #C3667, 0.2U/mL for two hours at 37°C; slides for versican, biglycan and fibromodulin). Slides were washed and then incubated (4°C overnight) with affinity-purified rabbit polyclonal antibodies to the G1 region of aggrecan (provided by Dr John Mort, McGill University, Canada[28], 1:1000), the GAG-alpha and GAG-beta regions of versican (Millipore AB1032 and AB1033, respectively, both at 5μg/mL), and the human C-terminal peptide sequences of biglycan and fibromodulin (EF2 and EF3 respectively, generated by Dr Emily Fuller, University of Sydney, both at 1μg/mL). Antibody binding was detected with rabbit Envision (Dako, NovaRed) for 30 minutes at room temperature then counterstained with Mayer’s heamatoxylin.

### Statistical analysis and data visualisation

Stata SE version 12 was used for all analyses and data visualisations. Based on our previous studies in sheep tendon, a sample size of six was determined to be sufficient to detect a two-fold change in gene expression with a power of 80–98%, depending on the gene[14]. Gene expression data were not normally distributed so both the expression and the score data were analyzed using the nonparametric ranked Kruskal-Wallis analysis, followed by Mann-Whitney U (for unpaired data) and Wilcoxon (for paired data) tests in the first instance. Non-operated control and sham-operated tendons were not different for any histologic or gene expression parameters at 6 weeks post-operation, so these data were pooled as controls, although we acknowledge that the shams and NOCs were not independent, being right and left legs of the same horses. These tendons were, however, processed independently.

Results are presented as box plots with the median marked and hinges at the 25^th^ and 75^th^ percentile. Box plots are positioned adjacent to a SDFT graphic with significant changes induced by surgery indicated by shading that region. The graphs of results on the lateral side (left) are presented with the origin on the right hand side for symmetry. Gene expression results are presented on a logarithmic scale as the relative fluorescent units per microgram starting RNA to avoid issues with unexpected regulation of housekeeping genes[29,30].

A mixed model was used to determine whether surgery and any of the positional covariates (medial/lateral, proximal/distal, distance from midmetacarpal) was a significant factor in the change in expression for each gene, after logarithmic conversion to normalize the data distribution. The mixed model clustered the data by horse, limb (relevant for sham/NOC) and position (carpal/MCP) to adjust for random grouping errors. Significant differences (*P* < 0.05) in these covariates were determined from the log coefficients in models of each gene in both the full dataset (with surgery as a covariate) and in control SDFT only.

Associations between histology scores and gene expression were determined by generating partial correlation coefficients, using Kendall’s tau-b. This nonparametric process uses pairwise ranked data values between the two variables under study (ordinal scores and continuous untransformed fluorescence for gene expression) and thus does not require data to be normally distributed or the relationship between the variables to be linear. The subprogram “parttau” (written by James Fiedler and Alan H. Feiveson, Johnson Space Center, Houston, Tx, USA) was used within Stata to correct for confounders (surgery and positional location of original sample within the tendon). The Bonferroni correction was applied to the *P* values of the gene associations for each score, resulting in *P* < 0.003 being considered significant.

## Results

### Clinical findings

All horses recovered uneventfully, and were fully weight-bearing on the operated limb immediately following surgery. There was no swelling in the sham-operated limbs. There was palpable swelling associated with the surgical site in the horses with the partially transacted SDFT that extended approximately 3cm proximal and distal to the surgical incision. This swelling gradually became firmer over the six weeks following surgery.

Sham-operated and non-operated control tendons showed normal echogenicity and cross-sectional area prior to surgery and at all time-points after surgery. The lesion created by partial transaction of the SDFT was clearly visible by ultrasound in the mid-metacarpus at two, four and six weeks after surgery in all six partially transected tendons (Fig 1C). The ultrasound changes were milder with distance from the transected region of the tendon. By six weeks post-transection, the SDFT 6cm distal to the lesion showed normal echogenicity but increased cross-sectional area in all hemi-transected tendons. The SDFT 6cm proximal to the lesion showed normal echogenicity in three of the partially transected tendons with the other three showed areas of hypo-echogenicity. At 8cm proximal and distal to the site of transection, all tendons appeared normal by ultrasound at all post-operative times.

### Gross morphology

Lesions created by partial transection had distracted to create a V-shaped area filled with a red-tinged fibrous tissue that extended beyond the normal margins of the tendon (Fig 1B). Pink discoloration and tissue softness was apparent throughout the length of the isolated tendon. In contrast, both sham-operated and non-operated control tendons appeared normal, being off-white in colour and firm on palpation (Fig 1B inset).

### Histopathology

**Fig 2:**
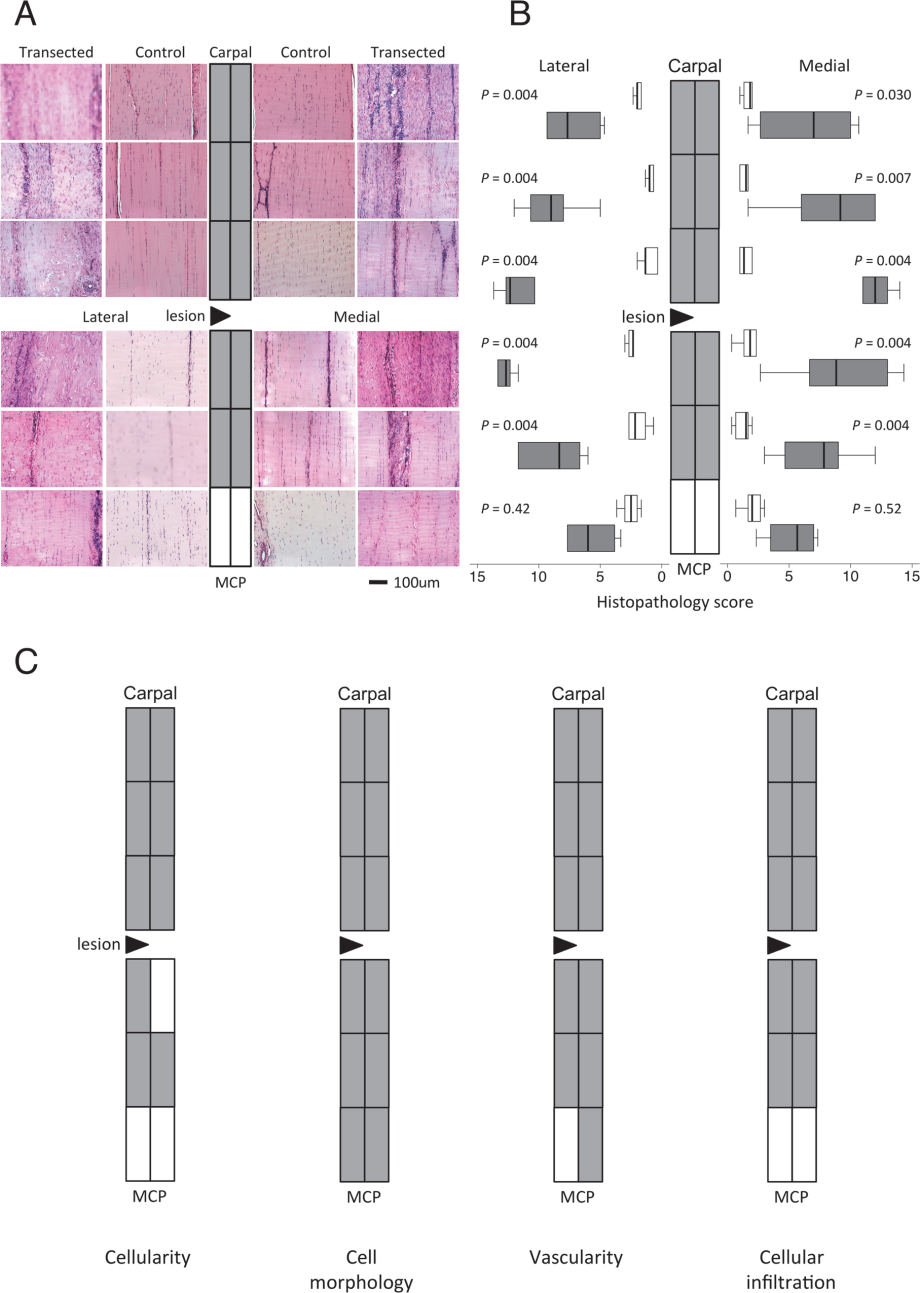
Tendon histopathology. Representative light microscopic images of haematoxylin and eosin-stained sections (A) from each location from a control and transected SDFT mapped to a diagram of the SDFT as depicted in Fig 1A. (B) Topographically-mapped box plot of overall histopathology scores ((n = 6 per group and region) of partially transected tendons (dark bars) compared with control SDFT (light bars). The lateral lesion site in the transected tendons is indicated by a triangle. As the horizontal scale indicates, expression on lateral side increases from right to left for display symmetry. Tendon regions in the central diagram are shaded if the score difference between control and transected tendons (indicated *P* values) is significant at the 5% level by Mann-Whitney U. Overall histopathology scores were higher in regions near the site of transection compared to regions further away in the partially transected tendons (*P* < 0.009, Kruskal-Wallis analyses). There were no significant differences in histological parameters between medial and lateral halves of the tendons. Lines within the boxes represent the median, the boxes represent the 25th and 75th percentiles, and the lines outside the boxes correspond to the minimum and maximum values. C) Topographical maps of significant changes in the indicated histopathology scores. Full box plots for these scores are in S2 Fig.

**Fig 3:**
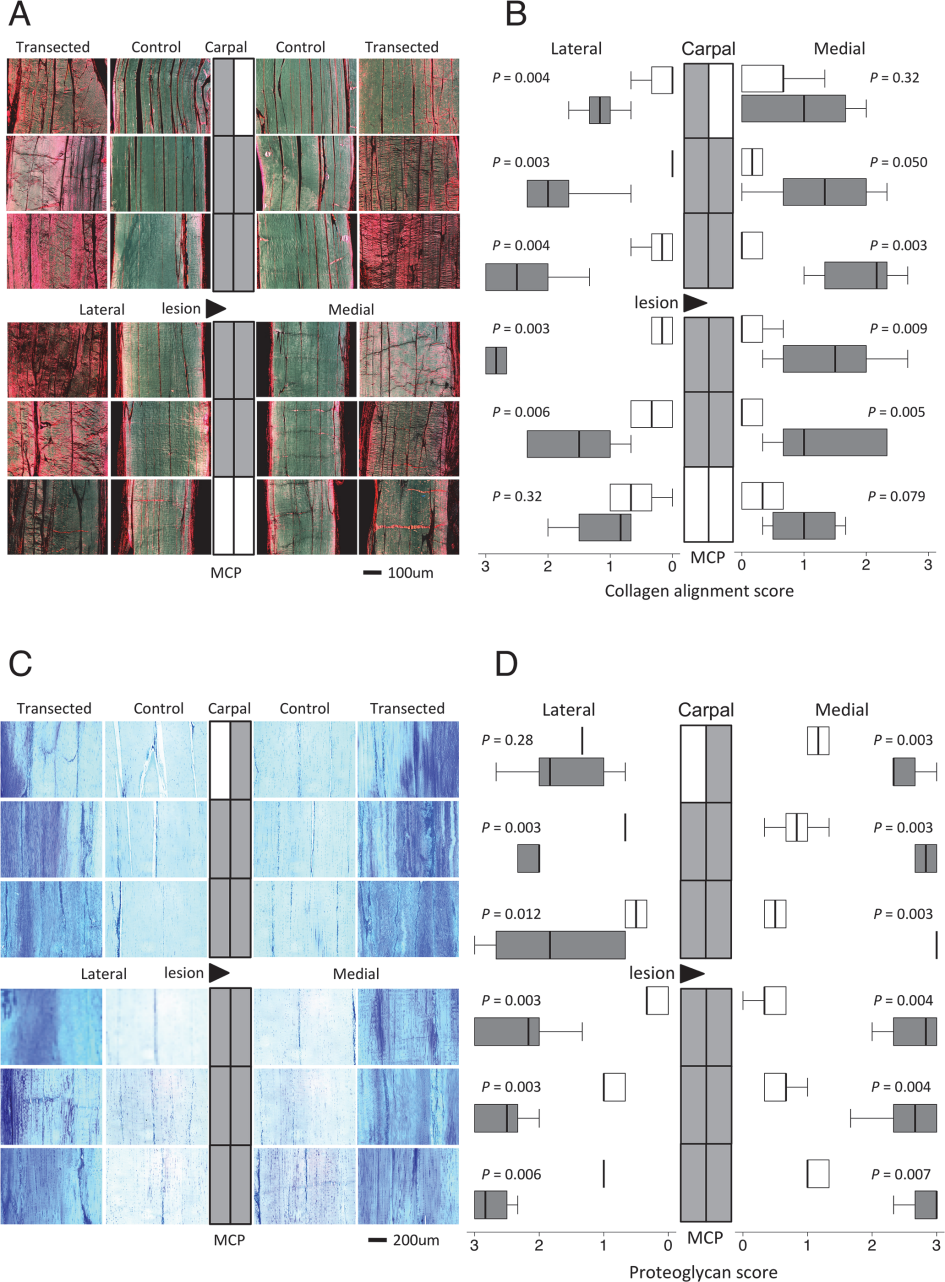
Collagen alignment and proteoglycan scores. Representative microscopic images of (A) picrosirius red-stained sections (polarised light) and (C) toluidine blue-stained sections (normal light) from each location from a control and transected SDFT mapped to a diagram of the SDFT. Topographically-mapped box plots of (B) collagen fiber alignment scores and (D) proteoglycan scores of partially transected tendons (dark bars) compared with control SDFT (light bars). The lateral lesion site in the transected tendons is indicated by a triangle. As indicated on the horizontal logarithmic scale, expression on lateral side increases from right to left for display symmetry. Tendon regions in the central diagram are shaded if the score difference between control and transected tendons (indicated *P* values) is significant at the 5% level by Mann-Whitney U.

At six weeks post surgery, there were no differences in the histology scores between non-operated control and sham-operated tendons. Data from these SDFT were pooled as the control group. Representative topographically mapped images of the H&E and PSR-stained sections of tendon are presented in Fig 2A and 3A respectively. All of the histological parameters were significantly increased in 9–12 regions of the partially transected compared with control tendons (Fig 2B and 2C, S1 Fig). The overall histopathology scores were significantly higher in the partially transected tendons compared to the control tendons in all regions (*P* < 0.02), except for the most distal (Fig 2B) and were higher in regions near the site of transection compared to regions further away (*P* < 0.009). Importantly, there were no significant differences found in histological parameters between medial and lateral halves of the tendons despite the presumed differences in loading resulting from the hemi-transection (over-stressed and stress-deprived for medial and lateral halves respectively). Hemi-transection decreased collagen fiber alignment with the magnitude of change being greater with proximity to the lesion but not different between medial and lateral halves (Fig 3A and 3B).

The control tendons showed regional variation in proteoglycan content with inter-fascicular metachromatic staining near bony attachments but little in the mid-metacarpal regions (Fig 3C and 3D). The proteoglycan content was greatly increased in partially transected compared with control tendon (*P* < 0.020) in all but the most proximal lateral region (Fig 3D). There were no significant differences between the lateral and medial halves or distance from the lesion of partially transacted tendons.

### Gene expression by real time RT-PCR

**Fig 4:**
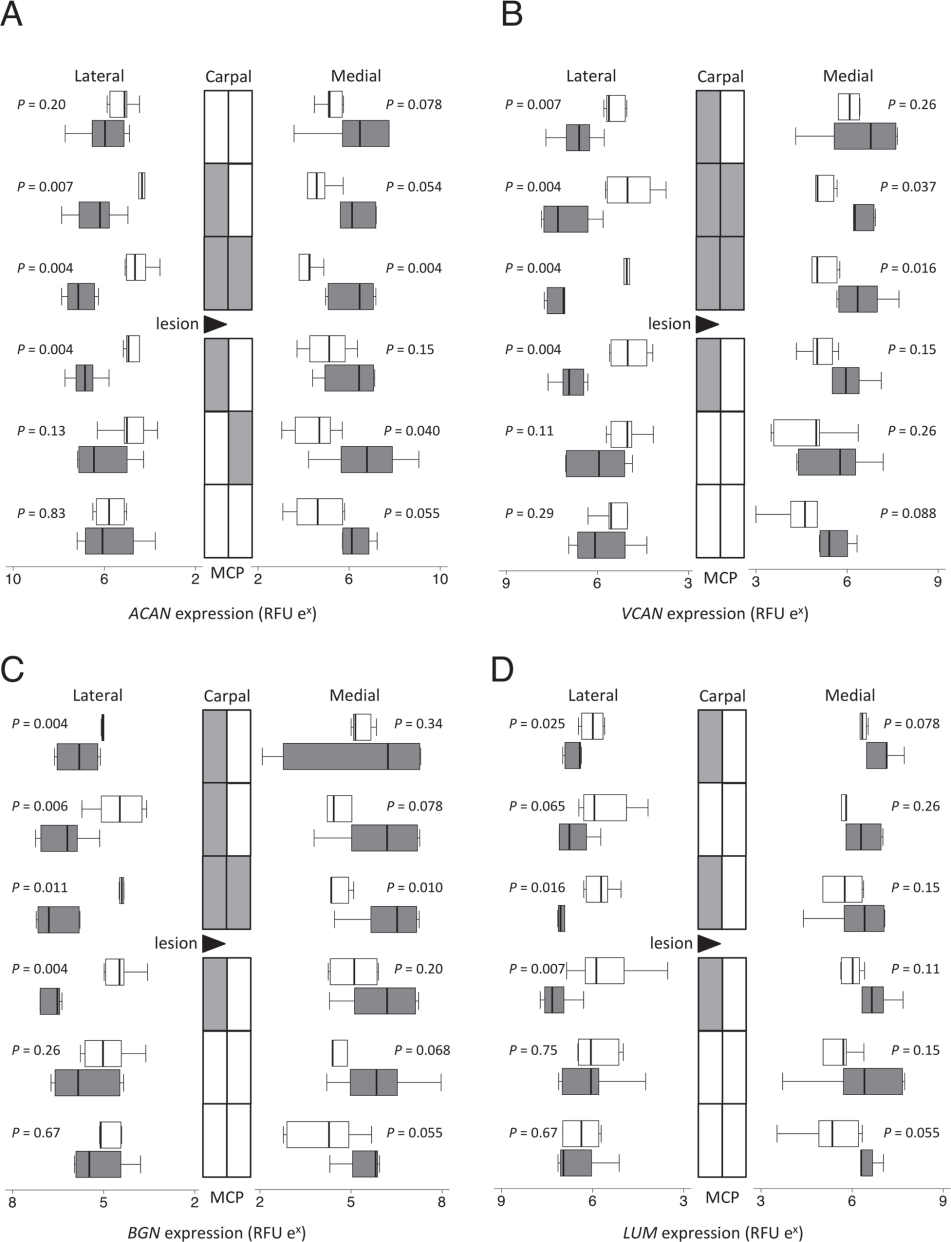
Proteoglycan gene expression. Topographically-mapped box plots (n = 6 per group and region) of (A) aggrecan, (B) versican, (C) biglycan and (D) lumican gene expression by partially transected tendons (dark bars) compared with control SDFT (light bars). The lateral lesion site in the transected tendons is indicated by the black triangle. As the horizontal logarithmic scale indicates, expression on lateral side increases from right to left for display symmetry. Tendon regions in the central diagram are shaded if the score difference between control and transected tendons (indicated *P* values) is significant at the 5% level by Mann-Whitney U. RFU = relative fluorescent units. Differences in expression between different locations as assessed by mixed model regression are summarized in Table 2.

**Fig 5:**
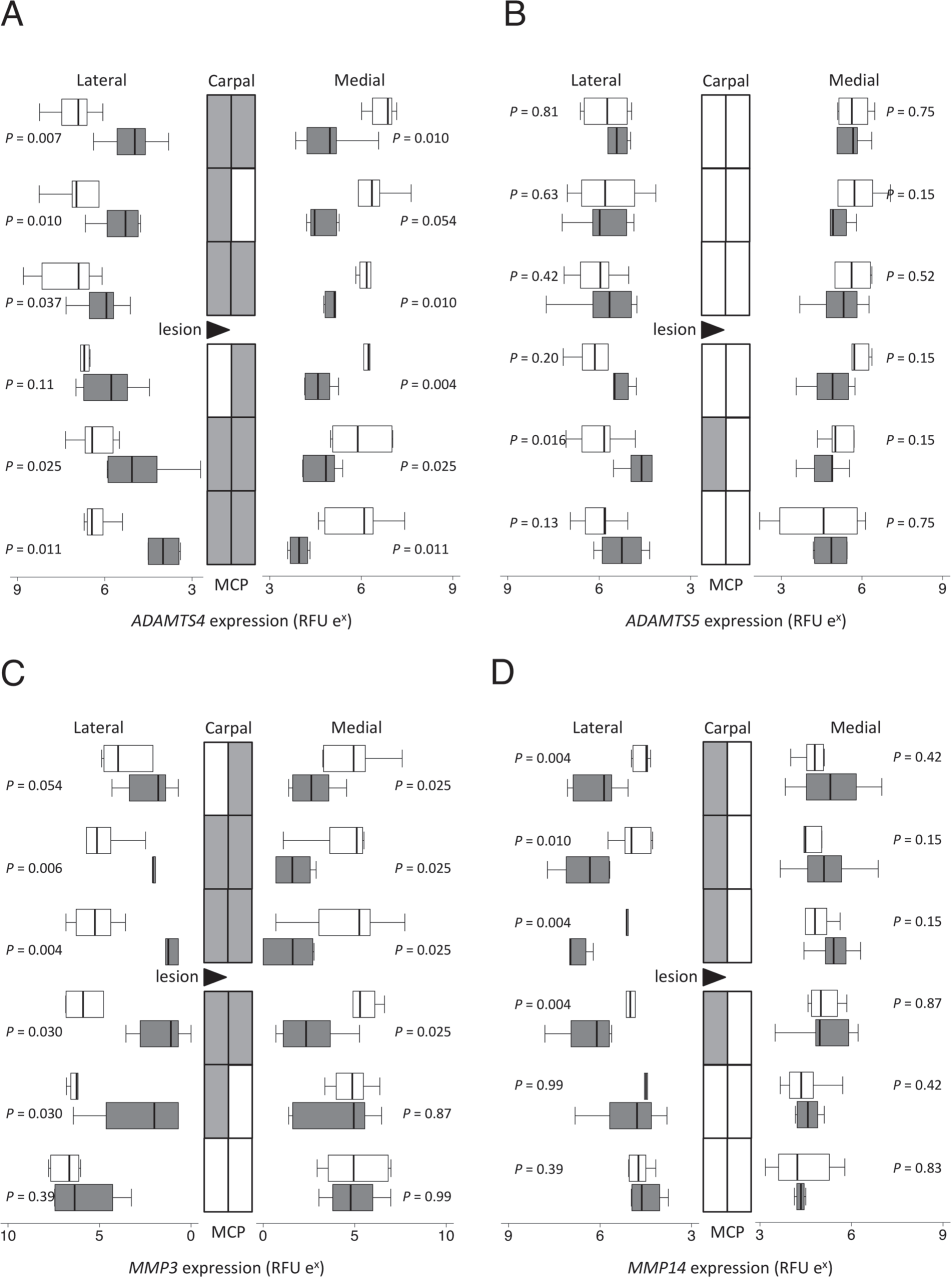
Proteinase gene expression. Topographically-mapped box plots of (A) *ADAMTS4*, (B) *ADAMTS5*, (C) *MMP3* and (D) *MMP14* gene expression (n = 6 per group and region) by partially transected tendons (dark bars) compared with control SDFT (light bars). The lateral lesion site in the transected tendons is indicated by the black triangle. As the horizontal logarithmic scale indicates, expression on lateral side increases from right to left for display symmetry. Tendon regions in the central diagram are shaded if the score difference between control and transected tendons (indicated *P* values) is significant at the 5% level by Mann-Whitney U. RFU = relative fluorescent units. Differences in expression between different locations as assessed by mixed model regression are summarized in Table 2.

**Fig 6:**
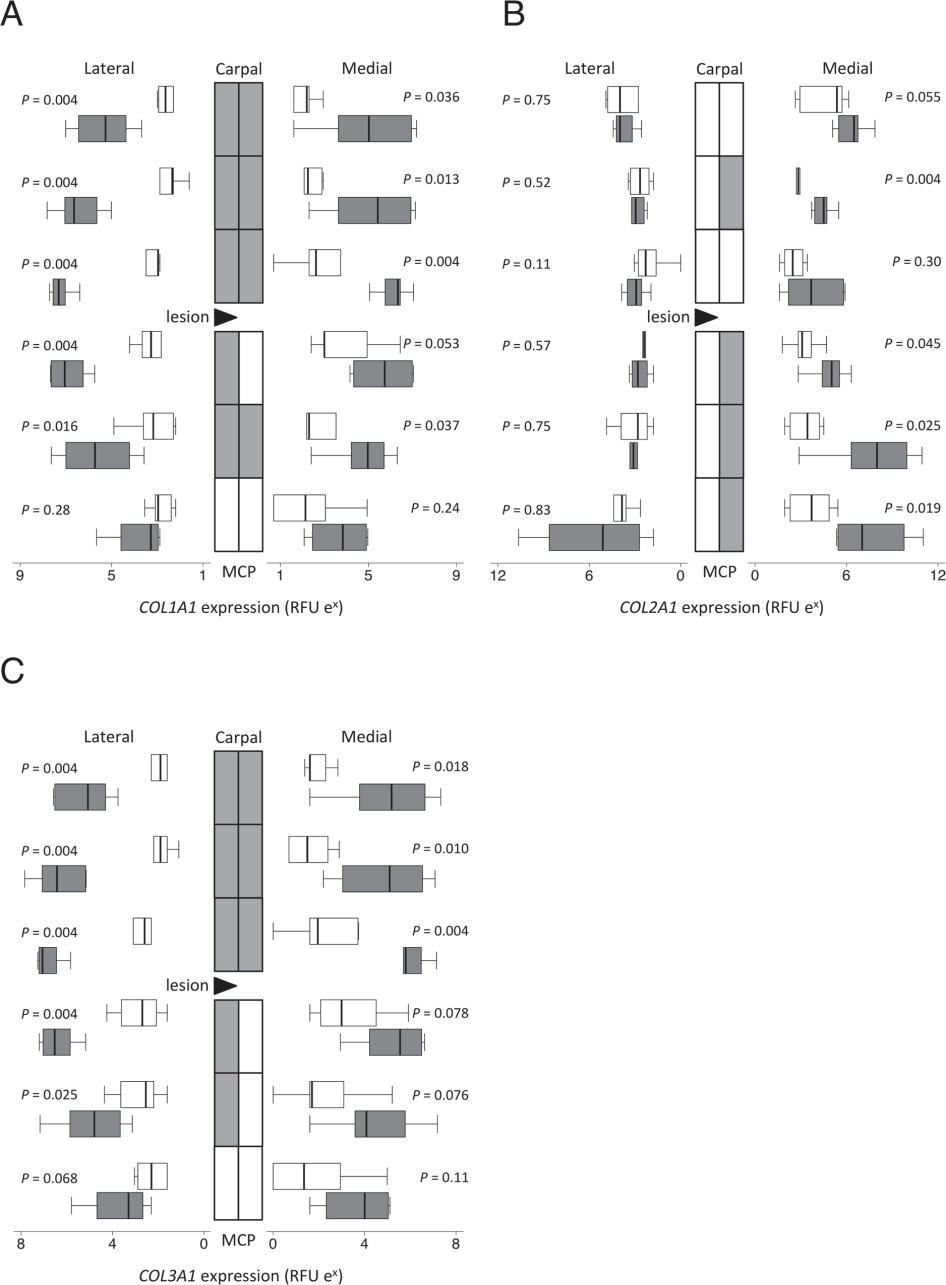
Collagen gene expression. Topographically-mapped box plots of the alpha-1 chains of collagen types I (*COL1A1*; A); II (*COL2A1*; B) and III (*COL3A1*; C) gene expression (n = 6 per group and region) by partially transected tendons (dark bars) compared with control SDFT (light bars). The lateral lesion site in the transected tendons is indicated by a triangle. As the horizontal logarithmic scale indicates, expression on lateral side increases from right to left for display symmetry. Tendon regions in the central diagram are shaded if the score difference between control and transected tendons (indicated *P* values) is significant at the 5% level by Mann-Whitney U. D) RFU = relative fluorescent units. Differences in expression between different locations as assessed by mixed model regression are summarized in Table 2.

As there were no significant differences in expression for any of the genes measured between non-operated control and sham-operated SDFT, these data were combined as the control group. Gene expression data are presented as topographically-mapped box plots in Figs 4–6 and the direction of change and significances from mixed model analyses of effect of surgery and regional variations are presented in Table 2. Model-generated estimates of beta coefficients (with 95% confidence intervals) are given as supplementary data (S1 Table). The coefficients are not reiterated in the text below as the actual relative fluorescent unit values are arbitrary but fold differences between control and transected samples are given where appropriate.

**Table 2:** Mixed regression modelling of gene expression data.

		Covariates			
Gene	*P* for model	Transection surgery	SideLateral (L) Medial (M)	Proximity to C or MCP	Distance from midmetacarpus /lesion site
***Controls only***					
*ACAN*	0.021		nd (0.38)	nd (0.22)	Increase (0.006)
*COL1A1*	0.002		nd (0.07)	nd (0.09)	Decrease (0.004)
*COL2A1*	<0.001		nd (0.30)	nd (0.29)	Increase (<.001)
*COL3A1*	0.005		nd (0.14)	nd (0.17)	Decrease (0.004)
*MMP3*	0.003		nd (0.10)	MCP > C (0.001)	nd (0.91)
*ADAMTS4*	<0.001		L > M (<.001)	C > MCP (0.033)	nd (0.35)
*ADAMTS5*	0.005		L > M (0.001)	nd (0.37)	nd (0.52)
***Controls and operated***					
*ACAN*	0.002	Increase (<.001)	nd (0.26)	nd (0.22)	nd (0.22)
*VCAN*	<.001	Increase (<.001)	L > M (0.002)	C > MCP (0.010)	nd (0.63)
*BGN*	0.004	Increase (0.001)	nd (0.23)	nd (0.79)	nd (0.12)
*FMOD*	0.066	- (0.011)	nd (0.19)	nd (0.47)	nd (0.93)
*LUM*	0.012	Increase (0.005)	L > M (0.038)	nd (0.75)	nd (0.34)
*DCN*	0.11	nd (0.06)	nd (0.49)	nd (0.67)	nd (0.08)
*COMP*	0.72	nd (0.58)	nd (0.94)	nd (0.97)	nd (0.19)
*COL1A1*	<.001	Increase (<.001)	L > M (<.001)	nd (0.66)	Decrease (<.001)
*COL2A1*	<.001	Increase (0.004)	M > L (<.001)	MCP > C (0.003)	Increase (<.001)
*COL3A1*	<.001	Increase (<.001)	L > M (0.001)	nd (0.40)	Decrease (<.001)
*MMP3*	<.001	Decrease (<.001)	nd (0.75)	MCP > C (<.001)	Increase (0.001)
*MMP14*	<.001	Increase (0.016)	L > M (<.001)	C > MCP (0.008)	Decrease (<.001)
*ADAMTS4*	<.001	Decrease (<.001)	L > M (<.001)	C > MCP (<.001)	Decrease (<.001)
*ADAMTS5*	<.001	nd (0.29)	L > M (<.001)	C > MCP (0.026)	nd (0.49)
*TIMP1*	<.001	Increase (0.019)	L > M (<.001)	nd (0.05)	nd (0.06)
*TIMP2*	0.29	nd (0.36)	nd (0.24)	nd (0.10)	nd (0.92)
*TIMP3*	0.002	Decrease (<.001)	nd (0.95)	nd (0.62)	nd (0.06)
*GAPDH*	<.001	nd (0.66)	L > M (<.001)	C > MCP (0.007)	nd (0.75)

Data was clustered by horse, limb and proximity to carpus (C) or metacarpophalanges (MCP). Significant effects of surgery and SDFT spatial position as covariates (and resultant *P* values) were obtained with all indicated covariates included in the model. Results are given for significant models without transection (n = 72; controls only) and from all models with transection (n = 140). nd = no difference; C = carpus; MCP = metacarpophalangeal joint.

In control tendons, matrix gene expression was uniform throughout the SDFT except *ACAN* (*P* = 0.006) and *COL2A1* (*P* < 0.001), where expression was significantly less mid-metacarpus than towards the bone, and *COL1A1* and *COL3A1* (both *P* = 0.004), where expression was significantly more mid-metacarpus than towards the bone (Table 2). Both *ADAMTS4* and *ADAMTS5* genes were expressed more highly on the lateral than the medial side in control tendons (*P* ≤ 0.001).

As with the histopathology, there were gene expression changes throughout the tendon following injury, although some changes were topographically localized with respect to proximity of the lesion, proximal versus distal and/or side of tendon. Transection significantly increased *ACAN* mRNA up to 18 fold in the regions around the lesion site (*P* < 0.001; Fig 4A). Versican gene (*VCAN*) expression was increased significantly (up to 9 fold; *P* < 0.001) but with greater increases proximally (*P* = 0.010) and in the lateral regions (*P* = 0.002) (Fig 4B). Both biglycan (*BGN*; *P* = 0.001; Fig 4C) and lumican (*LUM*; Fig 4D) gene expression increased up to 4 fold with transection, higher in the lateral areas than the medial (*P* = 0.038). The gene expression of decorin (*DCN*; A in S2 Fig) and fibromodulin (*FMOD;* B in S2 Fig) did not change significantly with transection.

Expression of *ADAMTS4* (up to 10 fold; *P* <0.001; Fig 5A), but not *ADAMTS5* (*P* = 0.29; Fig 5B), significantly decreased throughout the SDFT after transection but more towards the attachments areas, such that *ADAMTS4* mRNA levels were significantly lower with distance from the mid-metacarpal (*P* < 0.001). *MMP3* gene expression was greater distally than near the carpus (Fig 5C) in both normal and transected SDFT. There was a significant reduction in *MMP3* mRNA around the lesion area (up to 70 fold; *P* < 0.001), with distance from the mid-metacarpus influencing expression levels (*P* = 0.001). On the other hand, *MMP14* increased focally only in lateral regions (up to 8 fold; *P* = 0.016; Fig 5D), resulting not only in higher levels in the lateral compared to medial (*P* < 0.001) but also differences in proximity to the carpus (*P* = 0.008) and decreasing expression with distance for the mid-metacarpus (*P* < 0.001). *MMP13* and *MMP1* expression were below limits of detection in all samples.

Expression of all three collagen alpha chain genes analyzed increased significantly with transection (up to 48, 370, and 70 fold for types I, II and III respectively; *P* < 0.005), with *COL1A1* and *COL3A1* expressed significantly more on the lateral side and *COL2A1* significantly more on the medial side (all *P* ≤ 0.001; Fig 6). After transection, *COL2A1* expression was higher distally than proximally (*P* = 0.003). The three TIMP genes measured had a uniform expression throughout the SDFT in control tendons (S3 Fig). After transection, *TIMP1* expression increased (up to 5 fold; *P* = 0.019; A in S3 Fig) more on the lateral than the medial side (*P* < 0.001), *TIMP2* mRNA overall was unchanged despite small focal decreases in some regions below the lesion (*P* = 0.36; B in S3 Fig) and *TIMP3* expression significantly decreased (up to 3 fold; *P* < 0.001; C in S3 Fig).

### Association studies and immunohistology

The tau-b coefficients and significance of associations between the histological scores and the gene expression results are given in Table 3. The gene expression of aggrecan, biglycan, fibromodulin and collagen type II alpha chain was significantly and positively associated with the proteoglycan score (*P* < 0.001). Increases in aggrecan, biglycan, fibromodulin, and collagen types I and III gene expression all positively correlated with histopathology scores (*P* < 0.001), whereas versican, lumican, *ADAMTS4* and *MMP14* expression positively correlated only with collagen fiber alignment scores (*P* < 0.001). Decreasing *MMP3* expression correlated uniquely with increasing cell number and cell rounding scores (*P* < 0.001).

**Table 3:** Partial correlations of histology score parameters and gene expression.

Gene	Proteoglycan score		Fiber alignment		Cellularity		Cell morphology		Vascularity	
	*tau*	*P*	*tau*	*P*	*tau*	*P*	*tau*	*P*	*tau*	*P*
*ACAN*	**0.200**	**<0.001**	**0.250**	**<0.001**	**0.239**	**<0.001**	**0.216**	**<0.001**	**0.181**	**0.001**
*VCAN*	0.124	0.007	**0.289**	**<0.001**	0.133	0.012	0.131	0.014	0.120	0.032
*BGN*	**0.192**	**<0.001**	**0.302**	**<0.001**	**0.259**	**<0.001**	**0.220**	**<0.001**	0.180	0.004
*FMOD*	**0.178**	**<0.001**	**0.241**	**<0.001**	**0.229**	**<0.001**	**0.192**	**<0.001**	0.164	0.006
*LUM*	0.111	0.016	**0.230**	**<0.001**	0.105	0.035	0.100	0.063	0.111	0.051
*DCN*	0.018	0.69	0.037	0.51	-0.027	0.60	0.008	0.87	0.005	0.92
*COMP*	0.058	0.25	0.048	0.39	0.089	0.086	0.062	0.15	0.011	0.84
*COL1A1*	0.082	0.086	**0.217**	**<0.001**	**0.281**	**<0.001**	**0.180**	**0.001**	**0.216**	**<0.001**
*COL2A1*	**0.186**	**<0.001**	0.113	0.021	-0.018	0.72	0.036	0.47	-0.005	0.91
*COL3A1*	0.094	0.056	**0.228**	**<0.001**	**0.254**	**<0.001**	**0.179**	**0.002**	**0.186**	**<0.001**
*MMP3*	-0.055	0.24	-0.057	0.32	**-0.278**	**<0.001**	**-0.209**	**<0.001**	-0.072	0.19
*MMP14*	0.006	0.89	**0.248**	**<0.001**	0.112	0.033	0.046	0.34	0.119	0.005
*ADAMTS4*	0.022	0.66	**0.159**	**<0.001**	0.024	0.65	0.047	0.33	0.022	0.64
*ADAMTS5*	-0.007	0.89	0.138	0.006	-0.015	0.78	0.002	0.96	0.048	0.30
*GAPDH*	0.007	0.87	0.114	0.029	0.030	0.55	0.039	0.36	0.029	0.56
*TIMP1*	0.078	0.080	**0.260**	**<0.001**	**0.242**	**<0.001**	0.151	0.003	0.138	0.013
*TIMP2*	0.001	0.98	0.142	0.006	0.056	0.28	0.018	0.70	0.082	0.10
*TIMP3*	0.074	0.075	0.083	0.12	-0.064	0.26	0.000	0.99	-0.033	0.58

Surgery and SDFT spacial position (lateral or medial; proximal or distal; distance from transection site) were included as confounders. Partial rank correlations are Kendall’s tau (see methods). *P*<0.003 was determined as significant (bold) after Bonferroni’s correction.

**Fig 7:**
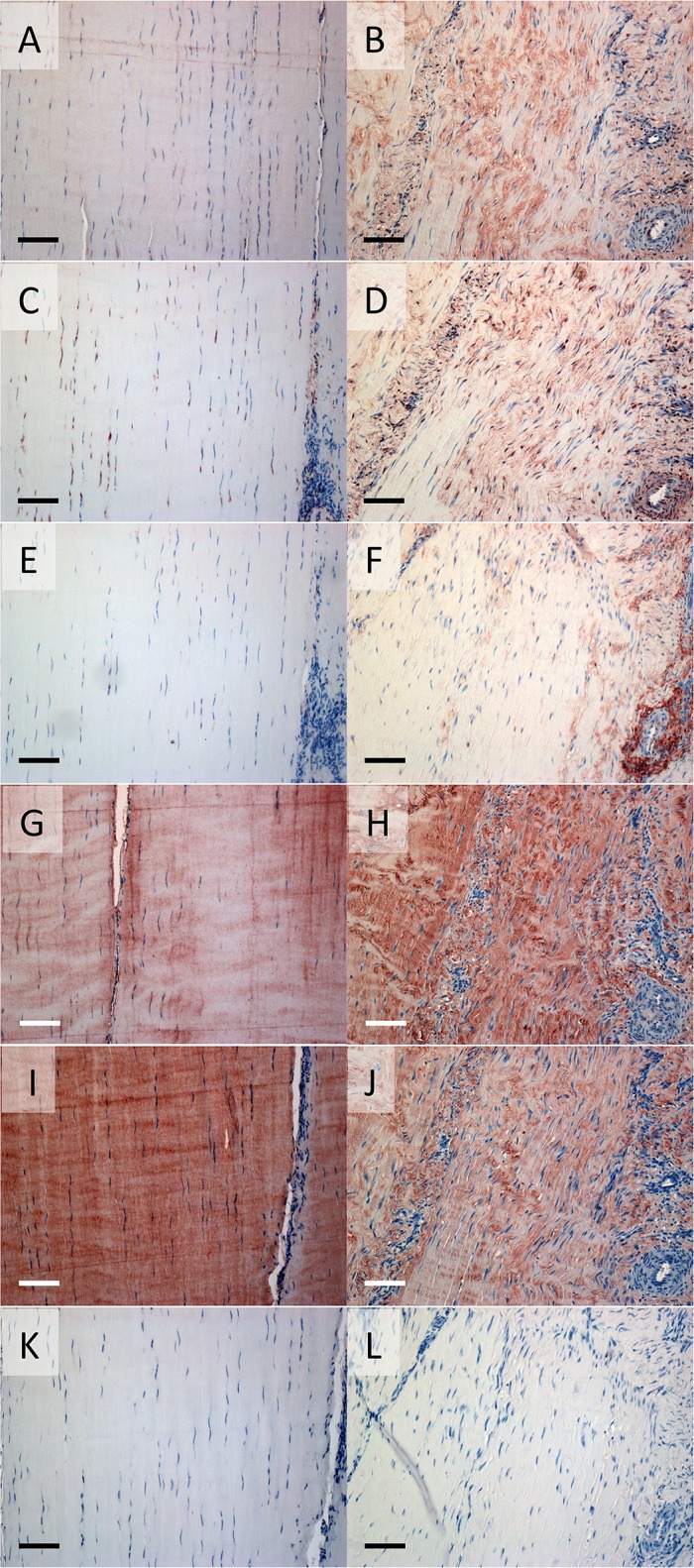
Tendon immunohistochemistry. Representative sections of a non-operated control (A,C,E,G,I,K) and transected stress-derived tendon (B,D,F,H,J,L) from the lateral side proximal to the lesion are shown. Positive immunostaining is seen as red/brown colour after incubating with antibodies to the aggrecan G1 domain (A,B); the versican GAG-alpha binding region (C,D); the versican GAG-beta binding region (E,F); the C-terminus of biglycan (G,H) and fibromodulin (I,J); and rabbit IgG (K,L; negative control). Scale bar indicates 0.1mm.

To validate that the gene expression data used for correlation with histopathology was reflective of protein levels, immunostaining was performed for several of the key proteoglycans (Fig 7). Consistent with PCR, staining for aggrecan, versican and biglycan, but not fibromodulin core proteins, was increased in transected tendons. Aggrecan staining was largely absent in control tendons, but was present in the intra- and inter-fascicular matrix following transection (Fig 7B). There was differential localisation of the GAG-alpha and GAG-beta containing isoforms of versican, with the latter being absent from control tendons while peri-cellular GAG-alpha was evident around tenocytes and inter-fascicular cells. In transected SDFT there was increased intra- and inter-fascicular cellular, matrix, and vascular GAG-alpha (Fig 7D), and GAG-beta staining became evident particularly in inter-fascicular regions around the blood vessels (Fig 7F). The intra-fascicular matrix of control tendons had diffuse staining for biglycan and fibromodulin (Fig 7G and 7I, respectively). While biglycan staining increased in intensity in transected tendons, fibromodulin was somewhat decreased (Fig 7H and 7J, respectively). Specificity of the various antibodies was demonstrated by lack of immunostaining in control or transected tendons with rabbit IgG (Fig 7K and 7L).

## Discussion

The equine SDFT provides a novel model to investigate the mechanisms of tendinopathy, its length allowing detailed regional analysis not readily available in other species[31–34]. Potential tendinopathy induction methods include overuse (e.g. forced treadmill running[35,36], chemical injections (e.g. collagenase[36]), or surgery (e.g. [14]). None of these models are perfect: overuse does not explain tendinopathy in the sedentary; exogenous collagenases elicit an acute widespread inflammatory response; and surgical transection is somewhat artificial for other than trauma-induced degeneration. Surgical models previously reported in the horse include a square, full thickness defect created by removing the central third of the equine SDFT[37] and an 8cm long core lesion in the equine SDFT created with a synovial resector[38], however these studies reported lesion size rather than changes in the adjacent tendon[26,36].

Ageing and exercise are known to induce matrix changes in the equine SDFT[27]. The horses used in this study were adult Standardbred geldings with an age range of 3–19 years. All horses were rested and had normal SDFTs prior to inclusion, however, pre-existing subclinical tendon damage could not be completely dismissed. The differences in composition between normal and diseased tendons rapidly became far greater than changes that occur with ageing[39,40]; therefore age-dependent variation was most likely minimal compared with that induced by tendon injury.

Little is known about the changes outside the immediate injury zone that result from focal tendon lesions. In an ovine model, degenerative histologic and gene expression changes were demonstrated throughout the infraspinatus tendon four weeks after 50% of the fibers were surgically transected at the mid point of the tendon[14]. The current study found similar histologic and gene expression changes extending at least 10cm proximal and distal to the hemi-transection lesion, well beyond detectable ultrasound abnormalities. This not only supports our hypothesis that widespread tendinopathy may explain the increased likelihood of future problems in tendons after injury[19–21] but also raises concerns about the sensitivity of ultrasound as a diagnostic technique. This equine SDFT model offers an excellent opportunity to test newer imaging techniques suggested to be superior in detecting tendon pathology, such as magnetic resonance imaging[41], ultrasound with tissue characterization[42], contrast-enhanced computed tomography[43], multi-detector computed tomography[44] and multimodal 2-photon microscopy[45].

That histologic and gene expression changes were significant and widespread six weeks after injury to the equine tendon, suggests studies that use tissue adjacent to a tear as the “normal comparator” for the lesion (e.g.[46–49]) should be interpreted with caution. We also identified regional variation in gene expression of ECM components (*ACAN*, *COL1A1*, *COL2A1*, *COL3A1*) and enzymes (*ADAMTS4*, *ADAMTS5*, *MMP3*) in the control equine SDFT. This is consistent with significant regional variation in gene expression described in the sheep infraspinatus[14] and human patellar tendons[50]. Thus care must be taken even when using normal tendons for comparative studies of tendinopathogenesis, to ensure that similar anatomical locations are matched. In our previous study of the infraspinatus tendon, regional variations could be explained by proximity to bone[14]. We postulate that, even within the long tensile equine SDFT well removed from bone and musculotendinous junction, the topographical differences are likely driven by specific biomechanical forces. It is interesting to speculate whether these underlying molecular differences may render particular areas more susceptible to degeneration and injury e.g. lower *ADAMTS* on the medial side.

The histopathological features in the tendinopathy distant from the hemi-transection site in equine SDFT, mirror those commonly reported in diseased human tendon[15,16,26,48,51], as do most of the gene expression changes: decreased *MMP3*[48,51–55] *and TIMP3*[52–55], and increased *MMP14*[48], *ACAN and BGN*[30,51,53,55], and *COL1A1* and *COL3A1*[48,51,53,55]. Expression of *MMP13* in human tendinopathy has been more variable, being increased in some cases[51,53,54] but undetectable or unchanged in others[48,52,53]. MMP13 upregulation may be associated with inflammation[25,36] and as an early response to unloading[14,56,57]. Variations in gene expression may also be related to location relative to the injury. The magnitude of the change in *COL1A1*, *COL3A1*, *ACAN*, *BGN* and MMP3 expression was greater near the lesion, suggesting that cellular/soluble factors (e.g. tumor necrosis factor-α[58,59]) released at the injury site acutely or during wound healing may play a greater role in regulation of these particular genes. In contrast, the restricted change in expression of *COL2A1* (medial only) and *MMP14* (lateral only) implicates a greater role for altered biomechanics (increased and decreased strain, respectively) in their regulation. The greater decrease in *ADAMTS4* distant from the lesion, and increased *VCAN* predominantly proximal, suggest additional complex regulatory control of injury-induced tendinopathy. Further work needs to be done to determine the exact contribution of mechanical cellular/soluble factors in the pathogenesis of tendinopathy. Nevertheless, it is clear that the underlying pathophysiology leads to subtle molecular differences with similar histologic outcomes. This has important implications when considering potential biological therapy of tendinopathy, particularly if administered locally.

Glycosaminoglycan accumulation is a classic feature of pathology in a variety of human tendons[26,48,50,60–64], and occurred throughout the length of the transected equine SDFT. The increased gene and protein expression of aggrecan and biglycan implicates these two proteoglycans, as previously suggested in human Achilles[30,65]. While increased[49,53], unchanged[30,48] and decreased[66] *VCAN* mRNA have been reported in human tendinopathy, versican protein content is routinely increased[50,60,61] as in the current equine model, and may also contribute to increased toluidine blue staining. The different isoforms of versican are independently regulated with tendon pathology[66], our results suggesting they are also differentially located within tendon. Decorin has longer glycosaminoglycan chains near the musculo- or osteo-tendinous junctions than mid-SDFT[67]. While we and others[30,48,50,68] found no tendinopathy-induced changes in *DCN* mRNA or core protein, post-translational modification of decorin or other proteoglycans, with longer glycosaminoglycans could also contribute to the increase in toluidine blue staining in tendinopathy.

Accumulation of proteoglycans in tendinopathy may result from an imbalance between expression/synthesis and removal. While loss of large proteoglycans was increased 2–3 fold in human tendinopathy, synthesis was increased 20–25 fold[61]. The widespread decrease in *ADAMTS4* gene expression (and by inference activity) reported here and previously[14], could contribute to reduced proteoglycan turnover. While best described as an aggrecanase, ADAMTS4 also degrades versican, biglycan, decorin, and fibromodulin[69]. Changes in *ADAMTS4* expression have not been observed in chronic human tendinopathies[51,52,61,70] while *ADAMTS5* mRNA is generally decreased[51–53]. The current and previously identified[14] decrease in *ADAMTS4* may be a consequence of the acute nature of the tendinopathy and/or of particular importance for injury-induced pathology. Nevertheless, approaches to maintain ADAMTS proteoglycanase activity may be a valid therapeutic target to avoid/manage tendon degeneration after injury.

We demonstrated parallel gene and protein changes in our model for a number of the key proteoglycans implicated in tendon pathology. While discordance between other mRNA and protein levels must be borne in mind, particularly enzyme activity that is also regulated through variable activation and inhibition, the associations between histopathology scores and gene expression results potentially provide some novel insights into the pathophysiology of injury-induced tendinopathy. Notably *ACAN* and *COL2A1* correlated significantly with toluidine blue staining, suggesting this is indicative of a bona fide chondroid metaplasia in diseased tendon. Increasing *LUM* and *VCAN* expression were significantly associated with changes in PSR staining but not other pathology variables, suggesting that changes in these two proteoglycans may have a particular effect on increasing collagen fiber malalignment. *DCN* expression did not correlate with any histopathology score, suggesting its well-known role in normal collagen fibrillogenesis[71] is not recapitulated in pathology or healing of tendon after injury. The negative association between *MMP3* expression and cellular changes (increasing numbers of more rounded cells as *MMP3* expression decreases) may suggest this enzyme plays a critical role in regulating cell migration and morphology in tendon.

In conclusion, this study has demonstrated that tendon injury is not a focal disease, but rather is associated with widespread tendinopathy. The rapid (within 6 weeks) extensive changes included proteoglycan accumulation and altered expression of matrix proteins, proteinases and inhibitors that are likely to have detrimental effects on regional tendon biomechanics and contribute to the poor outcome seen in clinical cases. Preliminary biomechanical testing of transected and control SDFT has indicated widespread change and we are determining associations between the resultant viscoelastic data and histologic and gene expression results in the present study (*manuscript in preparation*). Further studies should aim to identify histologic and gene expression changes at more time-points following injury to better understand how the healing environment changes over time and whether the observed widespread chondroid metaplasia of the injured tendon resolves. By increasing the knowledge of the molecular changes associated with the development of widespread tendon pathology, we may discover new targets for intervention therapies to prevent the debilitation due to injury and perhaps enable prevention of tendon injuries in the first place.

## Supporting Information

S1 Fig Tendon histopathology. Topographically-mapped box plots of (A) cellularity, (B) cell morphology, (C) interfasicular cell infiltration and (D) vascularity scores (n = 6 per region) of partially transected tendons (dark bars) compared with control SDFT (light bars). The lateral lesion site in the transected tendons is indicated by a triangle. As the horizontal scale indicates, scores on lateral side increase from right to left for display symmetry. Tendon regions in the central diagram are shaded if the score difference between control and transected tendons (indicated *P* values) is significant at the 5% level by Mann-Whitney U. There were no significant differences in histological parameters between medial and lateral or between proximal and distal halves of the tendons.
(TIF)

S2 Fig Proteoglycan and COMP gene expression. Topographically-mapped box plots of (A) *DCN*, (B) *FMOD*, and (C) *COMP* gene expression by partially transected tendons (dark bars) compared with control SDFT (light bars). The lateral lesion site in the transected tendons is indicated by the black triangle. As indicated on the horizontal logarithmic scale, expression on lateral side increases from right to left for display symmetry. Tendon regions in the central diagram are shaded if the score difference between control and transected tendons (indicated *P* values) is significant at the 5% level by Mann-Whitney U. RFU = relative fluorescent units. Differences in expression either after surgery or between different locations by mixed model regression are summarized in Table 2.
(TIF)

S3 Fig TIMP gene expression. Topographically-mapped box plots of expression (n = 6 per group and region) of *TIMP1* (A), *TIMP2* (B), *TIMP3* (C) by partially transected tendons (dark bars) compared with control SDFT (light bars). The lateral lesion site in the transected tendons is indicated by the black triangle. As the horizontal logarithmic scale indicates, expression on lateral side increases from right to left for display symmetry. Tendon regions in the central diagram are shaded if the score difference between control and transected tendons (indicated *P* values) is significant at the 5% level by Mann-Whitney U. RFU = relative fluorescent units. Differences in expression induced by surgery or between different locations as assessed by mixed model regression are summarized in Table 2.
(TIF)

S1 File Excel file of raw data including gene expression relative fluorescent units and histopathology scores for all samples. (XLS)

S1 Table Beta coefficients and P values for mixed models performed on gene expression data (summarised in Table 2). b = beta co-efficient; CI = confidence interval; L/M = lateral/medial; C/MCP = carpal/metacarpophalangeal.
(DOCX)
