# Correction: Focal Experimental Injury Leads to Widespread Gene Expression and Histologic Changes in Equine Flexor Tendons

**DOI:** 10.1371/journal.pone.0150823

**Published:** 2016-03-01

**Authors:** Else Jacobson, Andrew J. Dart, Takamitsu Mondori, Neil Horadogoda, Leo B. Jeffcott, Christopher B. Little, Margaret M. Smith

The first author’s name is spelled incorrectly. The correct name is Else Jacobson. The correct citation is: Jacobson E, Dart AJ, Mondori T, Horadogoda N, Jeffcott LB, Little CB, et al. (2015) Focal Experimental Injury Leads to Widespread Gene Expression and Histologic Changes in Equine Flexor Tendons. PLoS ONE 10(4): e0122220. doi:10.1371/journal.pone.0122220
