# Supplementary figures and images for: Focal Experimental Injury Leads to Widespread Gene Expression and Histologic Changes in Equine Flexor Tendons
Source: PLoS One. 2015 Apr 2;10(4):e0122220. doi: 10.1371/journal.pone.0122220 (PMC4383631; doi:10.1371/journal.pone.0122220)

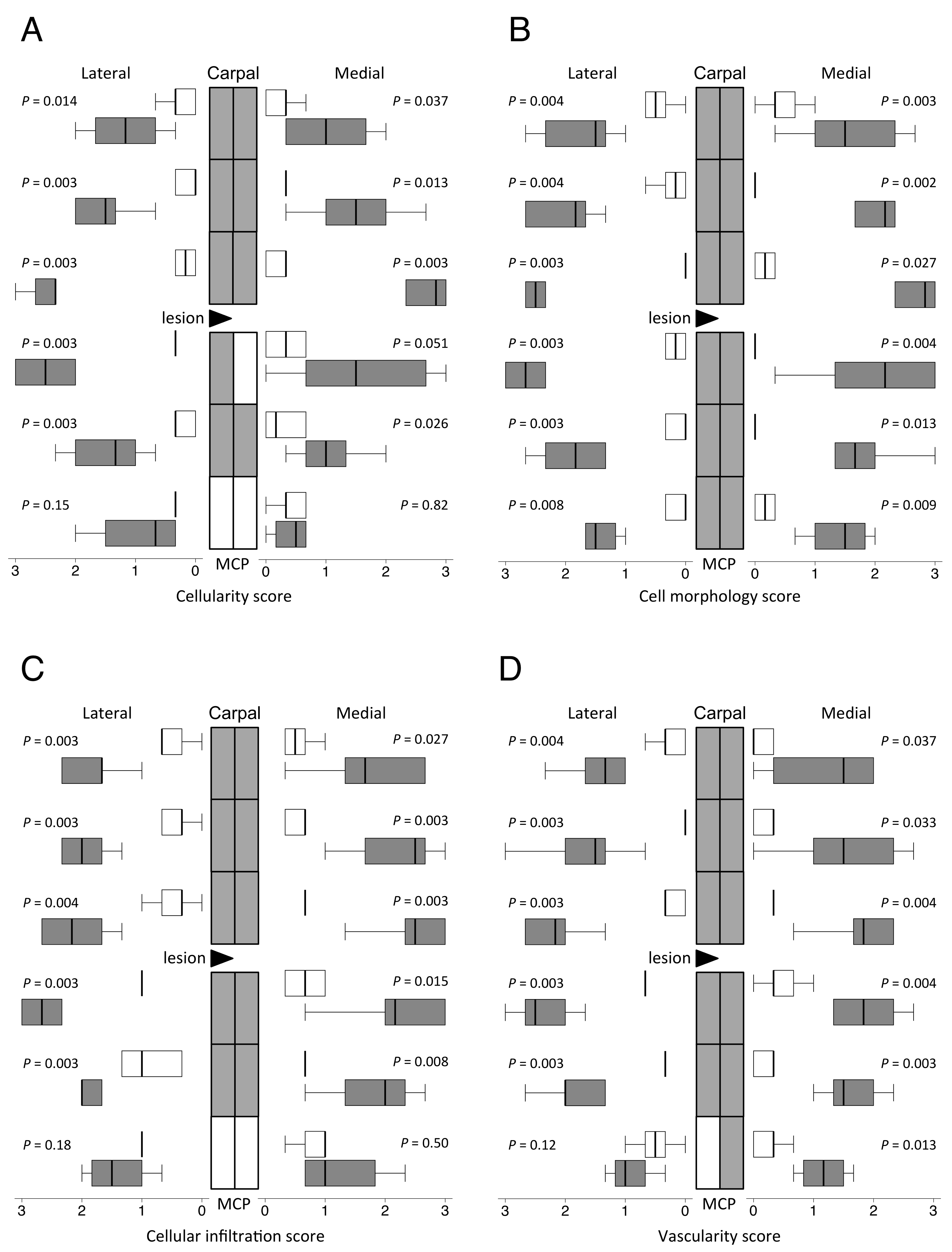

Supplement: S1 Fig — Topographically-mapped box plots of (A) cellularity, (B) cell morphology, (C) interfasicular cell infiltration and (D) vascularity scores (n = 6 per region) of partially transected tendons (dark bars) compared with control SDFT (light bars). The lateral lesion site in the transected tendons is indicated by a triangle. As the horizontal scale indicates, scores on lateral side increase from right to left for display symmetry. Tendon regions in the central diagram are shaded if the score difference between control and transected tendons (indicated P values) is significant at the 5% level by Mann-Whitney U. There were no significant differences in histological parameters between medial and lateral or between proximal and distal halves of the tendons. (TIF) [file pone.0122220.s001.tif]

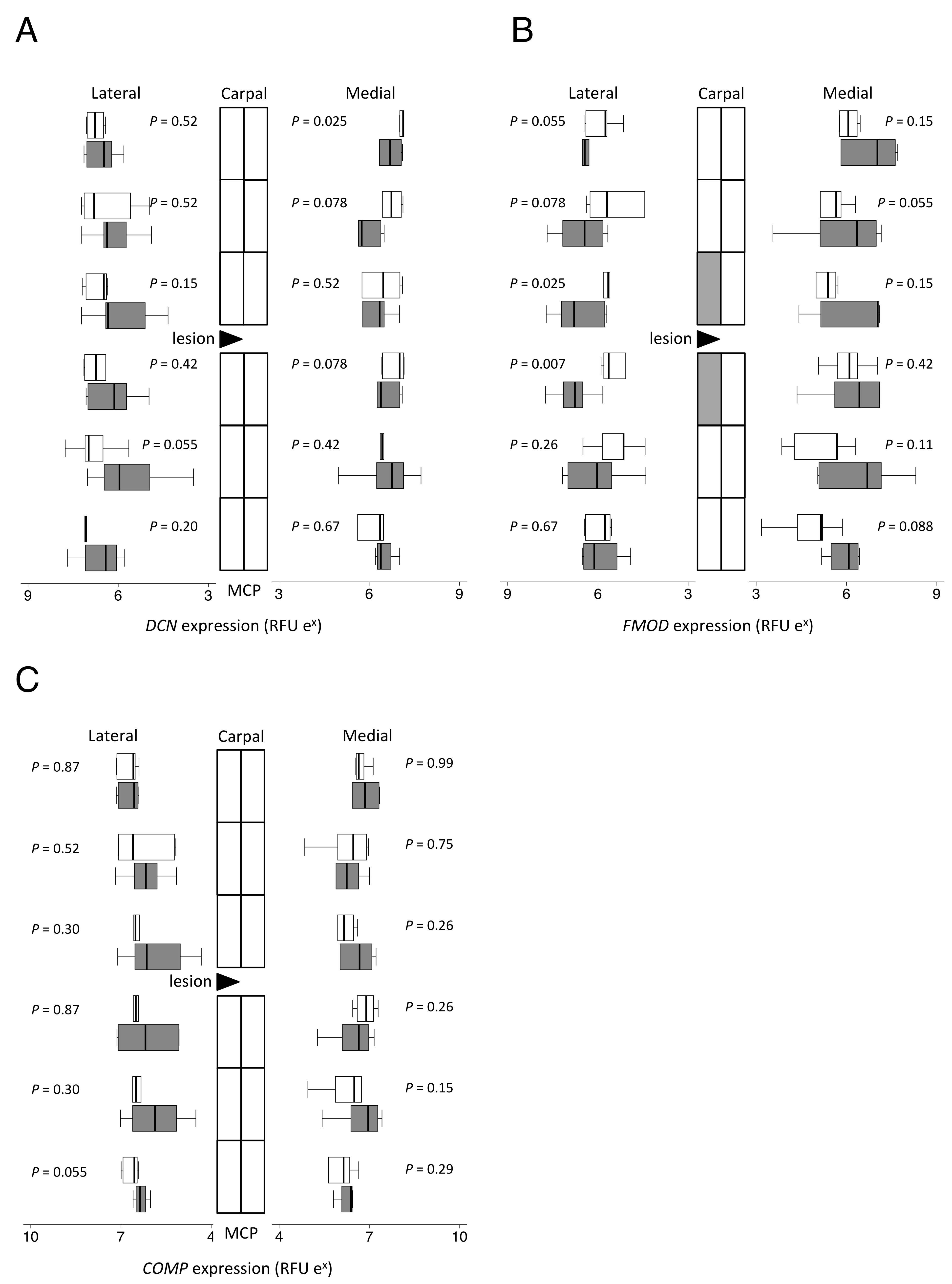

Supplement: S2 Fig — Topographically-mapped box plots of (A) DCN, (B) FMOD, and (C) COMP gene expression by partially transected tendons (dark bars) compared with control SDFT (light bars). The lateral lesion site in the transected tendons is indicated by the black triangle. As indicated on the horizontal logarithmic scale, expression on lateral side increases from right to left for display symmetry. Tendon regions in the central diagram are shaded if the score difference between control and transected tendons (indicated P values) is significant at the 5% level by Mann-Whitney U. RFU = relative fluorescent units. Differences in expression either after surgery or between different locations by mixed model regression are summarized in Table 2. (TIF) [file pone.0122220.s002.tif]

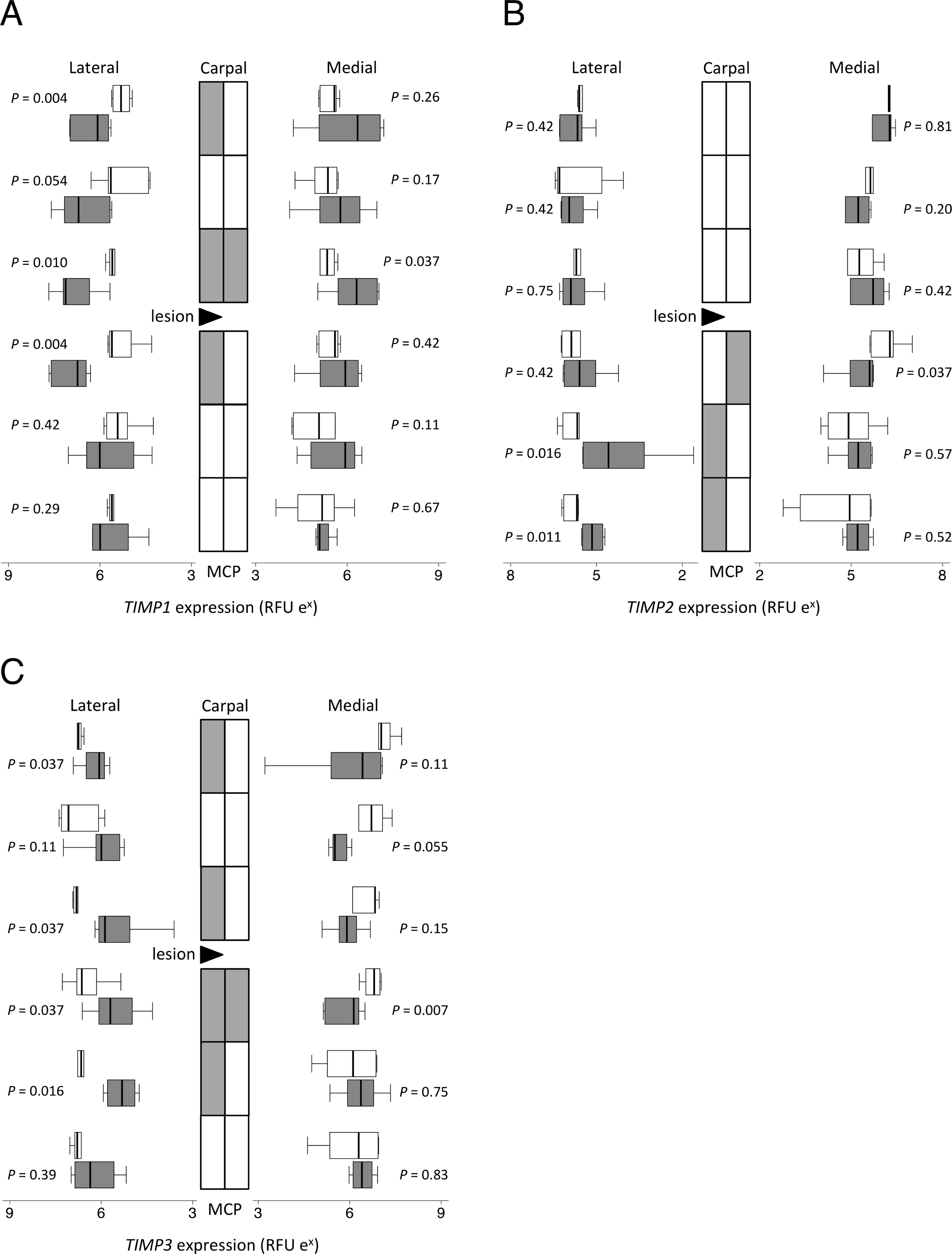

Supplement: S3 Fig — Topographically-mapped box plots of expression (n = 6 per group and region) of TIMP1 (A), TIMP2 (B), TIMP3 (C) by partially transected tendons (dark bars) compared with control SDFT (light bars). The lateral lesion site in the transected tendons is indicated by the black triangle. As the horizontal logarithmic scale indicates, expression on lateral side increases from right to left for display symmetry. Tendon regions in the central diagram are shaded if the score difference between control and transected tendons (indicated P values) is significant at the 5% level by Mann-Whitney U. RFU = relative fluorescent units. Differences in expression induced by surgery or between different locations as assessed by mixed model regression are summarized in Table 2. (TIF) [file pone.0122220.s003.tif]
