# Supplementary material for: Focal Experimental Injury Leads to Widespread Gene Expression and Histologic Changes in Equine Flexor Tendons
Source: PLoS One. 2015 Apr 2;10(4):e0122220. doi: 10.1371/journal.pone.0122220 (PMC4383631; doi:10.1371/journal.pone.0122220)
Supplement: S1 Table — b = beta co-efficient; CI = confidence interval; L/M = lateral/medial; C/MCP = carpal/metacarpophalangeal. (DOCX) [file pone.0122220.s005.docx]

**Table 2S. Beta coefficients and P values for mixed models performed on gene expression data** (summarised in Table 2).

|  | Model | Surgery | | | |  | L/M | | |  | C/MCP | | |  | Distance from lesion | | |  |
| --- | --- | --- | --- | --- | --- | --- | --- | --- | --- | --- | --- | --- | --- | --- | --- | --- | --- | --- |
| Gene | P | *b* | Lower 95% CI | Upper 95% CI | *P* | | *b* | Lower 95% CI | Upper 95% CI | *P* | *b* | Lower 95% CI | Upper 95% CI | P | *b* | Lower 95% CI | Upper 95% CI | P |
|  |  | Not included (controls only) | | |  | |  |  |  |  |  |  |  |  |  |  |  |  |
| *ACAN* | 0.021 |  |  |  |  | | 0.176 | -0.214 | 0.566 | 0.38 | -0.274 | -0.710 | 0.162 | 0.22 | **0.111** | **0.031** | **0.191** | **0.006** |
| *COL1A1* | 0.002 |  |  |  |  | | 0.413 | -0.026 | 0.852 | 0.065 | -0.419 | -0.913 | 0.075 | 0.096 | **-0.133** | **-0.223** | **-0.043** | **0.004** |
| *COL2A1* | <0.001 |  |  |  |  | | -0.243 | -0.699 | 0.213 | 0.30 | -0.305 | -0.866 | 0.255 | 0.29 | **0.261** | **0.168** | **0.354** | **<0.001** |
| *COL3A1* | 0.005 |  |  |  |  | | 0.355 | -0.115 | 0.826 | 0.14 | -0.399 | -0.968 | 0.169 | 0.17 | **-0.143** | **-0.239** | **-0.047** | **0.004** |
| *MMP3* | 0.003 |  |  |  |  | | 0.467 | -0.090 | 1.023 | 0.10 | **-0.934** | **-1.491** | **-0.378** | **0.001** | 0.006 | -0.107 | 0.120 | 0.91 |
| *ADAMTS4* | <0.001 |  |  |  |  | | **0.584** | **0.316** | **0.851** | **<0.001** | **0.477** | **0.038** | **0.916** | **0.033** | -0.026 | -0.081 | 0.029 | 0.35 |
| *ADAMTS5* | 0.005 |  |  |  |  | | **0.720** | **0.304** | **1.136** | **0.001** | 0.191 | -0.224 | 0.607 | 0.37 | -0.028 | -0.113 | 0.057 | 0.52 |
|  |  | Controls and transected | | |  | |  |  |  |  |  |  |  |  |  |  |  |  |
| *ACAN* | 0.002 | **1.633** | **0.744** | **2.523** | **<0.001** | | 0.164 | -0.123 | 0.451 | 0.26 | -0.208 | -0.558 | 0.142 | 0.22 | 0.037 | -0.022 | 0.097 | 0.22 |
| *VCAN* | <0.001 | **1.288** | **0.579** | **1.997** | **<0.001** | | **0.407** | **0.155** | **0.659** | **0.002** | **0.356** | **0.086** | **0.625** | **0.010** | 0.013 | -0.039 | 0.065 | 0.63 |
| *BGN* | 0.004 | **1.320** | **0.545** | **2.095** | **0.001** | | 0.169 | -0.107 | 0.445 | 0.23 | 0.038 | -0.239 | 0.315 | 0.79 | -0.045 | -0.102 | 0.012 | 0.12 |
| *FMOD* | 0.066 | 0.843 | 0.195 | 1.490 | 0.011 | | 0.187 | -0.092 | 0.465 | 0.19 | 0.103 | -0.176 | 0.382 | 0.47 | 0.003 | -0.054 | 0.060 | 0.93 |
| *LUM* | 0.012 | **0.769** | **0.227** | **1.310** | **0.005** | | **0.308** | **0.017** | **0.599** | **0.038** | -0.458 | -0.338 | 0.246 | 0.75 | 0.029 | -0.031 | 0.090 | 0.34 |
| *DCN* | 0.11 |  |  |  | 0.058 | |  |  |  | 0.49 |  |  |  | 0.67 |  |  |  | 0.084 |
| *COMP* | 0.72 |  |  |  | 0.58 | |  |  |  | 0.94 |  |  |  | 0.97 |  |  |  | 0.19 |
| *COL1A1* | <0.001 | **2.752** | **1.902** | **3.602** | **<0.001** | | **0.643** | **0.324** | **0.962** | **<0.001** | 0.091 | -0.313 | 0.462 | 0.66 | **-0.214** | **-0.280** | **-0.148** | **<0.001** |
| *COL2A1* | <0.001 | **1.511** | **0.486** | **2.535** | **0.004** | | **-1.195** | **-1.659** | **-0.731** | **<0.001** | **-0.745** | **-1.246** | **-0.246** | **0.003** | **0.316** | **0.220** | **0.412** | **<0.001** |
| *COL3A1* | <0.001 | **2.933** | **1.972** | **3.895** | **<0.001** | | **0.558** | **0.224** | **0.893** | **0.001** | 0.198 | -0.267 | 0.663 | 0.40 | **-0.191** | **-0.261** | **-0.122** | **<0.001** |
| *MMP3* | <0.001 | **-2.383** | **-3.481** | **-1.286** | **<0.001** | | 0.076 | -0.383 | 0.535 | 0.75 | **-1.210** | **-1.671** | **-0.749** | **<0.001** | **0.160** | **0.066** | **0.255** | **0.001** |
| *MMP14* | <0.001 | **0.788** | **0.148** | **1.427** | **0.016** | | **0.586** | **0.374** | **0.799** | **<0.001** | **0.413** | **0.107** | **0.720** | **0.008** | **-0.910** | **-0.135** | **-0.047** | **<0.001** |
| *ADAMTS4* | <0.001 | **-1.502** | **-2.023** | **-0.982** | **<0.001** | | **0.570** | **0.368** | **0.772** | **<0.001** | **0.486** | **0.215** | **0.757** | **<0.001** | **-0.080** | **-0.122** | **-0.038** | **<0.001** |
| *ADAMTS5* | <0.001 | -0.343 | -0.984 | 0.297 | 0.29 | | **0.601** | **0.327** | **0.875** | **<0.001** | **0.357** | **0.042** | **0.672** | **0.026** | -0.020 | -0.076 | 0.037 | 0.49 |
| *TIMP1* | <0.001 | **0.806** | **0.134** | **1.478** | **0.019** | | **0.394** | **0.195** | **0.592** | **<0.001** | 0.196 | -0.003 | 0.396 | 0.054 | -0.039 | -0.080 | 0.002 | 0.063 |
| *TIMP2* | 0.29 |  |  |  | 0.36 | |  |  |  | 0.24 |  |  |  | 0.099 |  |  |  | 0.92 |
| *TIMP3* | 0.002 | **-0.642** | **-1.000** | **-0.284** | **<0.001** | | -0.007 | -0.239 | 0.225 | 0.95 | 0.070 | -0.206 | 0.346 | 0.62 | 0.046 | -0.002 | 0.094 | 0.058 |
| *GAPDH* | <0.001 | 0.082 | -0.288 | 0.452 | 0.66 | | **0.341** | **0.173** | **0.508** | **<0.001** | **0.233** | **0.065** | **0.401** | **0.007** | -0.006 | -0.040 | 0.029 | 0.75 |

*b* = beta co-efficient; CI = confidence interval; L/M = lateral/medial; C/MCP = carpal/metacarpophalangeal
